# The complete mitochondrial genome of the Antarctic springtail *Cryptopygus antarcticus *(Hexapoda: Collembola)

**DOI:** 10.1186/1471-2164-9-315

**Published:** 2008-07-01

**Authors:** Antonio Carapelli, Sara Comandi, Peter Convey, Francesco Nardi, Francesco Frati

**Affiliations:** 1 Department of Evolutionary Biology, University of Siena, Via A. Moro 2, 53100 Siena, Italy; 2 British Antarctic Survey, NERC, High Cross, Madingley Road, Cambridge CB3 OET, UK

## Abstract

**Background:**

Mitogenomics data, i.e. complete mitochondrial genome sequences, are popular molecular markers used for phylogenetic, phylogeographic and ecological studies in different animal lineages. Their comparative analysis has been used to shed light on the evolutionary history of given taxa and on the molecular processes that regulate the evolution of the mitochondrial genome. A considerable literature is available in the fields of invertebrate biochemical and ecophysiological adaptation to extreme environmental conditions, exemplified by those of the Antarctic. Nevertheless, limited molecular data are available from terrestrial Antarctic species, and this study represents the first attempt towards the description of a mitochondrial genome from one of the most widespread and common collembolan species of Antarctica.

**Results:**

In this study we describe the mitochondrial genome of the Antarctic collembolan *Cryptopygus antarcticus *Willem, 1901. The genome contains the standard set of 37 genes usually present in animal mtDNAs and a large non-coding fragment putatively corresponding to the region (A+T-rich) responsible for the control of replication and transcription. All genes are arranged in the gene order typical of Pancrustacea. Three additional short non-coding regions are present at gene junctions. Two of these are located in positions of abrupt shift of the coding polarity of genes oriented on opposite strands suggesting a role in the attenuation of the polycistronic mRNA transcription(s). In addition, remnants of an additional copy of *trnL(uag) *are present between *trnS(uga) *and *nad1*. Nucleotide composition is biased towards a high A% and T% (A+T = 70.9%), as typically found in hexapod mtDNAs. There is also a significant strand asymmetry, with the J-strand being more abundant in A and C. Within the A+T-rich region, some short sequence fragments appear to be similar (in position and primary sequence) to those involved in the origin of the N-strand replication of the *Drosophila *mtDNA.

**Conclusion:**

The mitochondrial genome of *C. antarcticus *shares several features with other pancrustacean genomes, although the presence of unusual non-coding regions is also suggestive of molecular rearrangements that probably occurred before the differentiation of major collembolan families. Closer examination of gene boundaries also confirms previous observations on the presence of unusual start and stop codons, and suggests a role for tRNA secondary structures as potential cleavage signals involved in the maturation of the primary transcript. Sequences potentially involved in the regulation of replication/transcription are present both in the A+T-rich region and in other areas of the genome. Their position is similar to that observed in a limited number of insect species, suggesting unique replication/transcription mechanisms for basal and derived hexapod lineages. This initial description and characterization of the mitochondrial genome of *C. antarcticus *will constitute the essential foundation prerequisite for investigations of the evolutionary history of one of the most speciose collembolan genera present in Antarctica and other localities of the Southern Hemisphere.

## Background

Mitochondria contain their own circular bacteria-like genome (mtDNA) that is physically separated from that of the cell nucleus. Metazoan mtDNA typically consists of a covalently closed circular molecule, tightly packed with a canonical set of 37 genes that encode for 13 inner membrane proteins, 2 ribosomal RNAs and 22 transfer RNAs. These genes have no introns and, generally, few intergenic spacers, the only exception being a large non-coding region (A+T-rich region in hexapods) where essential regulatory sequences involved in the initiation of replication and transcription have been identified [1,2]. The arrangement of genes along the mtDNA (gene order) is generally conserved within major taxonomic groups, but can differ extensively in specific lineages [3,4].

In the last few years, the genomic resources of public databases have accumulated a remarkable number of complete mitochondrial sequences for hexapods; these form the largest data set of mitogenomic data available for arthropods (59%: 81 of 151 sequences available in GenBank). Within Metazoa, the set of complete mtDNAs known from hexapod species is outnumbered only by a few vertebrate groups. Although numerically biased towards the pterygote orders, hexapod mtDNA data have been enriched recently with entries from the more basal lineages (apterygote), a possible consequence of the growing interest in phylogenetic studies of insects and relatives for the analysis of genome-level data sets [5-9]. At present, 10 complete (or almost complete) mtDNA sequences have been determined for collembolan (springtail) species, although detailed descriptions and analysis have been provided only for a minority. The "basic" arrangement of the 37 mitochondrial genes found in collembolan species generally resembles that usually considered ancestral for Pancrustacea [8,10]. However, rearrangements of tRNA genes and/or molecular traces of duplication events appear to be more common than expected.

Focusing on Antarctic organisms, a remarkable feature, the loss of *nad6*, has been reported for notothenioid fishes; this unusual rearrangement has been tentatively associated with heat production due to proton leakage, as a possible physiological adaptation to cold environments [11].

Collembola have been described as model organisms in Antarctic terrestrial ecosystem studies, on account of the extent of existing biochemical and ecophysiological research, and their dominant role in Antarctic terrestrial ecosystems [12]. In this study we describe the complete mitochondrial genome of the Antarctic collembolan *Cryptopygus antarcticus*, the first example from the family Isotomidae and only the second (after *Gomphiocephalus hodgsoni*; [5]) for an Antarctic species. Recently, molecular phylogeographic studies based on mitochondrial haplotypes have contributed to the understanding of the evolutionary origins of species of the genus *Cryptopygus *[13]. Mitogenomics data for *C. antarcticus *will further contribute to studies of the patterns of distribution and the processes of colonization of the Antarctic collembolan fauna, a subject of particular recent interest in the context of Antarctic phylogeography, carrying signals over multimillion year timescales [14,15].

## Results and Discussion

### Gene content and genome organization

**Figure 1:**
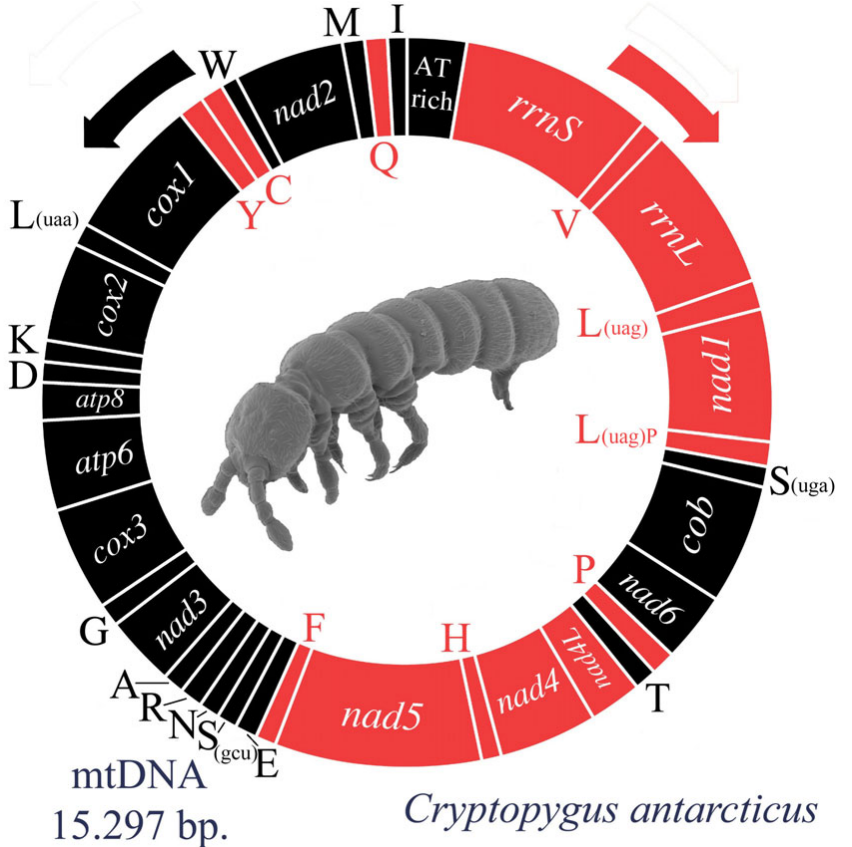
**The mitochondrial genome organization of *Cryptopygus antarcticus*.** Genes for proteins and rRNAs are indicated with standard abbreviations, whereas those for tRNAs are designated by a single letter for the corresponding amino acid. Arrows indicate direction of coding regions. Black color is used for the genes oriented on the J-strand, red for those with opposite polarity.

The mitochondrial genome of *C. antarcticus *is a closed circular molecule of 15,297 bp and contains the set of 37 genes usually found in metazoans [16] (Figure 1; GenBank accession number: EU016194). The gene order is identical to *Drosophila yakuba *[17], and reflects the presumed ancestral condition for Pancrustacea. The majority of genes are located on the *plus *or J-strand, the remainder having opposite polarity and being oriented on the *minus *or N-strand (Figure 1 & Table 1). There are four major non-coding regions of 622, 123, 60 and 33 bp in length that are located at the gene junctions *rrnS*/*trnI*, *trnS(uga)*/*cob, nad6/cob *and *trnE/trnF*, respectively. The largest non-coding region most likely corresponds to the region (A+T-rich region in insects) where in other hexapods the sequences responsible for the initiation of transcription and replication of the entire genome are found [1].

**Table 1:** Annotation, nucleotide composition and other features of the mitochondrial genome of *C. antarcticus*.

gene	strand	sites	seq position	%A	%C	%G	%T	%A+T	start codon	stop codon
*trnI*	J	62	Jan-62	33.9	11.3	16.1	38.7	72.6		
*trnQ*	N	68	64–131	33.8	10.3	19.1	36.8	70.6		
*trnM*	J	69	143–211	42.0	14.5	11.6	31.9	73.9		
*nad2*	J	997	214–1210	30.9	17.1	11.3	40.6	71.6	ATG(M)	T--
*trnW*	J	68	1211–1278	45.6	13.2	7.4	33.8	79.4		
*trnC*	N	62	1278–1339	35.5	12.9	16.1	35.5	70.9		
*trnY*	N	63	1363–1425	25.4	15.9	20.6	38.1	63.5		
*cox1*	J	1534	1429–2962	28.2	19.6	17.5	34.7	62.9	ATG(M)	T--
*trnL(uaa)*	J	64	2963–3026	37.5	10.9	15.6	35.9	73.4		
*cox2*	J	683	3027–3709	33.4	17.7	13.9	34.9	68.4	ATC(I)	TA-
*trnK*	J	71	3710–3780	32.4	21.1	18.3	28.2	60.6		
*trnD*	J	64	3781–3844	43.8	7.8	6.3	42.2	85.9		
*atp8*	J	165	3845–4009	35.8	19.4	6.7	38.2	73.9	ATC(I)	TAA
*atp6*	J	680	4003–4683	31.8	19.1	13.2	35.9	67.6	ATG(M)	TAA
*cox3*	J	789	4683–5471	30.7	19.1	15.7	34.5	65.1	ATG(M)	TAA
*trnG*	J	58	5494–5551	36.2	10.3	15.5	37.9	74.1		
*nad3*	J	343	3550–5892	34.1	18.1	11.7	36.2	70.3	ATA(M)	T--
*trnA*	J	61	5893–5953	36.1	9.8	16.4	37.7	73.8		
*trnR*	J	64	5953–6016	26.6	14.1	21.9	37.5	64.1		
*trnN*	J	64	6015–6078	34.4	14.1	18.8	32.8	67.2		
*trnS(gcu)*	J	67	6079–6145	31.3	14.9	16.4	37.3	68.7		
*trnE*	J	64	6147–6210	35.9	18.8	12.5	32.8	68.8		
*trnF*	N	62	6244–6305	37.1	11.3	19.4	32.3	69.4		
*nad5*	N	1706	6306–8011	27.4	12.1	16.2	44.3	71.7	ATA(M)	TA-
*trnH*	N	63	8009–8071	30.2	7.9	19.0	42.9	73.0		
*nad4*	N	1358	8072–9429	26.1	11.1	16.6	46.2	72.3	ATG(M)	TA-
*nad4L*	N	267	9436–9702	25.8	11.2	15.4	47.6	73.4	ATA(M)	TAA
*trnT*	J	62	9720–9781	43.5	9.7	11.3	35.5	79.0		
*trnP*	N	64	9782–9845	34.4	4.7	18.8	42.2	76.6		
*nad6*	J	495	9839–10333	36.6	12.5	10.1	40.8	77.4	ATT(I)	TAA
*cob*	J	1135	10394–11528	28.8	18.9	16.5	35.8	64.6	ATA(M)	T--
*trnS(uga)*	J	71	11529–11599	35.2	14.1	12.7	38.0	73.2		
*trnL(uag)P*	N	68	11654–11721	32.4	14.7	19.1	33.8	66.2		
*nad1*	N	928	11722–12649	23.2	11.9	20.7	44.2	67.3	TTA(L)	T--
*trnL(uag)*	N	63	12653–12715	33.3	9.5	14.3	42.9	76.2		
*rrnL*	N	1214	12716–13929	34.0	8.6	13.2	44.2	78.2		
*trnV*	N	61	13930–13990	27.9	11.5	21.3	39.3	67.2		
*rrnS*	N	685	13991–14675	34.5	9.1	14.0	42.5	76.9		
*A+T-rich*		622	14676–15297	45.8	5.6	9.9	38.6	84.4		
all mtDNA		15297		37.5	16.7	12.4	33.4	70.9		

### Gene initiation and termination

Canonical initiation codons (ATA or ATG), encoding the amino acid methionine, are used in 9 PCGs (*atp8, cob, cox1, cox3, nad2–5 *and *nad4L*), whereas four other genes start with non-standard codons (*atp6, cox2, nad1 *and *nad6*) (Table 1) as it often happens in animal mtDNAs [18]. Only five PCGs terminate with the complete termination codon TAA (*atp8, atp6, cox3, nad4L *and *nad6*). In all other cases, stop codons are truncated (T or TA) and their functionality probably recovered after a post-transcriptional polyadenilation [19]. These abbreviated stop codons are found in PCGs that are followed by a downstream tRNA gene, suggesting that the secondary structure information of the tRNA genes could be responsible for the correct cleavage of the polycistronic transcript. It is noteworthy that in all cases an additional complete stop codon is present a few bases downstream of the incomplete stop codon, as if a short overlap would be necessary to stop the RNA polymerase in case translation should begin before mRNA cleavage [3,20]. tRNA genes are usually interspersed among protein coding genes, their secondary structure acting as a signal for the cleavage of the polycistronic primary transcript [19,22]. However, there are also direct junctions between two PCGs (*atp8/atp6*, *atp6/cox3, nad6/cob *and *nad4L/nad4*) where other cleavage signals, different from tRNA gene secondary structures, may be involved in the processing of the polycistronic primary transcript [23]. In this respect, hairpin structures, frequently observed at the 3'-end of a PCG abutting the 5'-end of a neighbouring one, may serve as signal to direct immature mRNA cleavage [24-26]. Experimental analyses of cDNA pools have demonstrated that some mtDNA genes (i.e.: *atp8/atp6 *and *nad4L/nad4*) are recovered as bicistronic units in human HeLa cells [21] and in the dipteran *Anopheles funestus *[25]. In the *C. antarcticus *mtDNA, with the single exception of *cox3*, a complete stop codon (TAA) is only present when two PCGs abutt directly (Table 1). The gene pair *nad4L/nad4 *is composed of a single in-frame coding unit (the two genes are separated by 6 nucleotides), whereas *atp8 *and *atp6 *overlap (7 nucleotides) as is almost universally found in metazoans [25].

### Transfer RNAs

**Figure 2:**
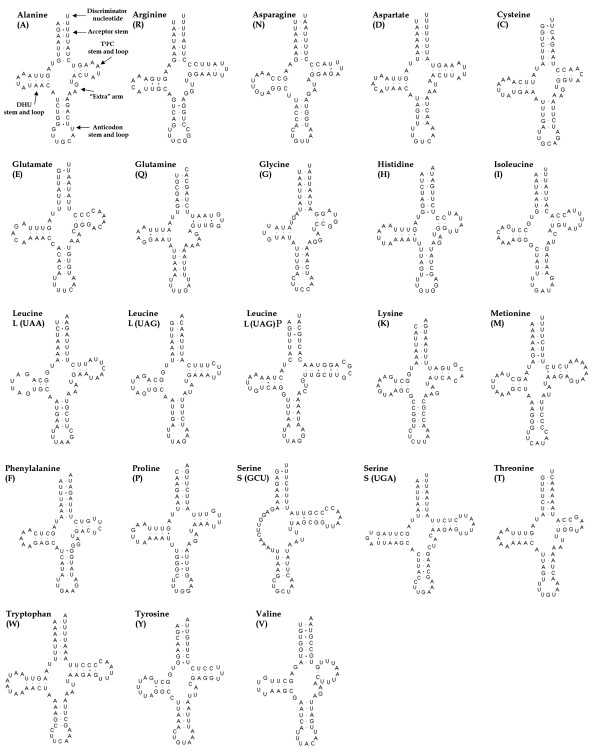
Putative secondary structures of tRNAs present in the mitochondrial genome of *C. antarcticus*.

All the 22 tRNA genes typically found in metazoan mtDNAs were identified according to their secondary structure and primary sequence of the corresponding anticodon (Figure 2). The only peculiar feature is represented by an additional (and apparently non-functional) copy of the tRNA gene for the amino acid leucine (*trnL(uag)P*), that is located within a non-coding spacer (123 bp) between *trnS(uga) *and *nad1 *(Figure 1). This tRNA pseudogene may represent the degenerating vestige of a duplication event that occurred in collembolan mtDNA early in the evolution of the group, given that in the same position a similar spacer is also present in *G. hodgsoni *[5] (50 bp) and *Podura aquatica *[8] (150 bp). Other unusual features are the lack of the DHU arm in *trnS(gcu) *and the reduction of the TΨC arm in *trnG *and *trnV *. Moreover, some tRNA genes have few mismatches in the acceptor and/or the discriminator arms (*trnR, trnD, trnC, trnE, trnQ, trnH, trnL(uaa), trnLuag, trnK, trnS(gcu) *and *trnW*). In similar cases (in metazoans but more commonly in plants and Protozoa) base pairing is restored post- or co-transcriptionally with an RNA-editing mechanism [27,28]. Overlaps between tRNA genes were not frequent (4 instances) and then limited to 1 or 2 bases (Table 1).

### Ribosomal RNAs

The putative secondary structures of the complete small and large ribosomal subunits (*rrnS *and *rrnL*) have been reconstructed for a limited number of insect species, and detailed studies are essentially available only for *Drosophila virilis*, *D. melanogaster *[29] and *Apis mellifera *[30]. Within basal hexapod groups, the architectures of the ribosomal subunits have been tentatively reconstructed only for the two species *Campodea fragilis *and *C. lubbocki *[31], and a more detailed analysis has been performed only for domain III of the small RNA subunit of several Arthropleona species [32]. Additional comparative analyses in basal hexapod lineages are required to allow an accurate comparison with current secondary structure models of holometabolan insects [30], and we therefore made no attempt to reconstruct the structure of the rrnS and rrnL rRNA here.

### A+T-rich region

Previous analyses of the *Drosophila *A+T-rich region identified the signals responsible for the origin of replication of the major and minor strands (O_J _and O_N_, respectively) and confirmed that, at least in this genus, the mtDNA replicates with a strand-asynchronous, asymmetric mechanism [1]. Mapping of O_J _and O_N _in *Drosophila *and other insect A+T-rich regions has recently allowed the definition of primary sequences and secondary structure elements involved in the initiation of the replication process [1,33-36]. In holometabolan insects, two distinct thymine stretches, one located near *trnI *on the N-strand and another in the center of the A+T-rich region on the J-strand, appear to be responsible for O_J _and O_N _replication, respectively. Similarly, a thymine stretch plays an essential role in the initiation of the replication system of the Light strand (O_L_) in mammals (although in this case the two O_R _are separated by 2/3 of the genome), and is also believed to be involved in protein binding and primer RNA synthesis. Nevertheless, the conservation of thymine stretches among insects appears to be a peculiarity of more derived orders, with alternative sequences probably responsible for O_R _in basal hexapod lineages [1].

The A+T-rich region of *C. antarcticus *is 622 bp; it shows six "TA" tandem repeats of variable length (from 5 to 7 units) and a low similarity in primary sequence with other collembolan specimens. The identification of sequences homologous to those observed in holometabolan insects involved in O_R _was not completely successful in defining potential regulatory elements. In this respect, four thymine stretches of variable size (4 to 6) could be observed on the N-strand, within 100 bp of *trnI*, but none of them appears to correspond, in terms of size and position, to that observed in *Drosophila*. In addition, in agreement with previous reports [1] there is no thymine stretch longer than 6 bp on this strand. Conversely, in the *C. antarcticus *J-strand, a longer thymine stretch (14 bp) is present between nucleotides 14996 and 15009 and this appears to be assimilable (both in primary sequence and position) to the one observed in the central portion of the *Drosophila *A+T-rich region. Similarly to *Drosophila*, this portion of the *C. antarcticus *A+T-rich region may also act as the O_N_, given that the motif "ACTATTT", frequently present in *Drosophila *(in 8/12 different species; see: Figure 6, in [1]), is found 11 bp downstream of the 14-bp thymine stretch. If this evidence is confirmed by additional data on basal hexapod mtDNA sequences, the role of conserved primary sequence stretches in the initiation of the replication mechanism might be confirmed as a common feature of all hexapod groups (at least for the N-strand), from the most basal to the most derived lineages. The presence of hypothetical secondary structures involved in the initiation of the origin of replication process of the *C. antarcticus *mtDNA is difficult to establish. In this species the entire A+T-rich region can be folded in several paired structures, and additional comparative studies will be needed to clarify their role.

### Non-coding regions

Unlike those of plants, animal mitochondrial genomes are very compact, with a high proportion of coding *vs *non-coding sequences [16,37]. Intergenic spacers are usually limited in number and size, and their occurrence is believed to be the result of errors of the mtDNA replication system (i.e. duplication of parts of the mitochondrial genomes, with redundant copies becoming pseudogenes and disappearing). Apart from the A+T-rich region, the largest (123 bp) non-coding sequence of the *C. antarcticus *mtDNA is located between *trnS(uga) *and *nad1*. Within this region, an additional copy of *trnL(uag) *(Figures. 1 and 2) can be identified. Given that a functional copy of this gene is present in its canonical position between *nad1 *and *rrnL *genes (as in other arthropod mtDNAs), and that there are several mismatches in the acceptor arm that might compromise the correct folding into a typical cloverleaf structure, we believe that *trnL(uag)P *may represent the molecular trace of an older duplication event that occurred during the evolution of this species (there are also alternative interpretations, see below). It is noteworthy that this genomic region is also subject to molecular rearrangments in other collembolan species (i.e. one tRNA translocation in *Tetrodontophora bielanensis *and *Onychiurus orientalis*) or shows intergenic spacers similar to those observed in *C. antarcticus *(i.e. 48 bp in *G. hodgsoni*). This observation suggests that the DNA fragment encompassing *cob *and *nad1 *may represent a "hot spot" for rearrangements in the collembolan mitochondrial genome, and that either several taxa have independently undergone rearrangements or these have occurred before the differentiation of the major collembolan lineages.

It is well known that tRNA genes are more prone to translocations than protein coding genes, with "hot spots" of rearrangement being described in some lineages [38,39]. In the case of the collembolan mtDNA, we speculate that a replication error (probably due to slipped strand mispairing) may have generated an intermediate state with at least a duplicated copy of the *trnL(uag) *gene. Afterwards, the superfluous copy(ies) may have been transformed into a pseudogene, whereas the original copy could have conserved its functionality (therefore leading to no changes in the gene order). Presently, it is only possible to conclude that: 1) similar (for size and position) intergenic spacers have been also found in two species of the genus *Campodea *(Diplura [31]), another basal hexapod group probably as primitive as Collembola; 2) the gene junction *trnS(uga)/nad1 *corresponds to the shift of the coding polarity between two blocks of genes (*nad6-cob-trnS(uga) *oriented on the J-strand, and *nad1-trnL(uag)-rrnL-trnV-rrnS *on the N-strand). Interestingly, experiments conducted on transcriptional termination factors in *D. melanogaster *have identified the positions of some binding sites that are responsible for the control of multiple transcription units on the mtDNA of this species [40]. One of the most interesting results of these studies is that, unlike human, and along with sea urchin mtDNA [41-44], in *Drosophila *one of these terminator sites is not located at the 3'-end of the ribosomal genes. Conversely, two stretches of non-coding sequence would correspond to the binding sites for the *Drosophila *mitochondrial transcription termination factor (DmTTF), a nuclear-encoded protein involved in the processes of attenuation and/or termination of mRNA transcripts [40]. Intriguingly, the short stretches of non-coding sequences identified in *Drosophila *as DmTTF binding sites are located at the gene junctions *trnE*/*trnF *and *trnS(uga)/nad1*, in the same positions where *C. antarcticus *shows intergenic spacers (Table 1). Not only their position is identical, but also these sequences are located in a position of abrupt shift in the transcription polarity of the mtDNA genes (a suitable position for an attenuator/terminator of mRNA synthesis). Nevertheless, the non-coding regions of *Drosophila *and *C. antarcticus*, show no discernible sign of homology, leaving open the question of whether they do play the same role. It should be noted, however, the signalling role may be played by secondary structure motifs, like those observed between *trnS(uga) *and *nad1 *in Collembola and Diplura mtDNAs, rather than primary sequence.

### Repeats and palindromic sequences

Two perfect palindromic sequences were observed. One is 38 bp long and spans the junction between the *nad2 *and *trnW *(positions 1196–1233). The second is 18 bp long and lies 42 bp from the 3'end of *rrnS *(14033–14050).

Three repeated sequences were found in different areas of the genome. One is 22 bp long and encompasses the initial part of both *trnL(uaa) *and *trnL(uag) *(note that these latter genes are oriented on opposite strands), from base 3 to base 24 (with one mismatch). Interestingly, although duplication and remolding events of leucine tRNA genes have probably occurred independently several times within some major animal lineages [45], the observed 22 bp-long fragment is highly conserved in all collembolan species investigated (data not shown) suggesting that primary sequence and precise conformation of this region is important for the building of both tRNA genes and/or for their interaction with the ribosomal complex.

A second perfect direct repeated sequence, 18 bp long, is observed at 105 bp from the 3'-end of *rrnS *(14096–14113), and at 7 bp from the 3'-end of *nad6 *(10309–10327). A partially overlapping sequence of 17 bp is also repeated in inverted orientation in the middle of the A+T-rich region.

### Base composition and codon usage

The mtDNA of many arthropod lineages is characterized by a strong compositional bias showing high values of A% and T% *vs*. G% and C%. This trend in nucleotide composition (A+T bias) is remarkable in some insect groups (i.e. holometabolan orders) and, to a lesser extent, is also apparent in the more basal hexapod lineages [31]. The *C. antarcticus *mtDNA is 70.9% A+T-rich, and therefore in the range observed for other collembolan species (A+T contents spanning from 65.5% to 74.1%) [5,8]. The observed A+T content strongly influences the use of two- and four-fold degenerate synonymous sites in protein coding genes, with relaxed pressure probably responsible for the most frequent use of NNA and NNU codons (Table 2).

**Table 2:** Relative synonymous codon usage in the protein-encoding genes of the mitochondrial genome of *C. antarcticus*.

Amino acid	codon	n	RSCU	Amino acid	codon	n	RSCU	Amino acid	codon	n	RSCU	Amino acid	codon	n	RSCU
Phe	UUU	303	1.72	Ser	UCU	114	2.61	Tyr	UAU	114	1.40	Cys	UGU	22	1.38
	UUC	50	0.28		UCC	19	0.43		UAC	49	0.60		UGC	10	0.62
Leu	UUA	286	3.31		UCA	73	1.67					Trp	UGA	65	1.44
	UUG	41	0.47		UCG	19	0.43						UGG	25	0.56
	CUU	88	1.02	Pro	CCU	54	1.58	His	CAU	43	1.23	Arg	CGU	15	1.07
	CUC	14	0.16		CCC	29	0.85		CAC	27	0.77		CGC	11	0.79
	CUA	77	0.89		CCA	41	1.20	Gln	CAA	57	1.75		CGA	22	1.57
	CUG	13	0.15		CCG	13	0.38		CAG	8	0.25		CGG	8	0.57
Ile	AUU	268	1.63	Thr	ACU	73	1.51	Asn	AAU	116	1.41	Ser	AGU	29	0.66
	AUC	60	0.37		ACC	34	0.70		AAC	48	0.59		AGC	9	0.21
Met	AUA	216	1.69		ACA	81	1.68	Lys	AAA	80	1.65		AGA	82	1.87
	AUG	40	0.31		ACG	5	0.10		AAG	17	0.35		AGG	5	0.11
Val	GUU	91	1.56	Ala	GCU	86	1.69	Asp	GAU	41	1.24	Gly	GGU	60	1.04
	GUC	16	0.27		GCC	45	0.89		GAC	25	0.76		GGC	17	0.3
	GUA	94	1.61		GCA	46	0.91	Glu	GAA	53	1.33		GGA	64	1.11
	GUG	32	0.55		GCG	26	0.51		GAG	27	0.68		GGG	89	1.55

Another remarkable molecular feature of metazoan mtDNAs is the asymmetry in the composition of the nucleotide content between the two strands [46]. Usually, A% and C% are higher than T% and G% on the J-strand (appropriately named *plus *strand because of its positive AT and CG skew), whereas the reverse is observed in the N-strand (hence named *minus *strand). Asymmetry in nucleotide composition among strands may be due to the peculiar replication and transcription mechanisms [47]. In mammals, it has been demonstrated that the duplication of the mitochondrial genome (usually defined as the 'strand-displacement' or 'leading and lagging' model) is asynchronous and requires extensive displacement of the H strand, that therefore transiently is found in a single-strand state ([48]; but see also alternative views in [49]). The longer the time this strand is unpaired during replication (D_SSH_), the more pronounced is the compositional asymmetry between H and L strands [50]. In the few cases where mtDNA replication has been studied in insects (essentially *Drosophila*), the mechanism is also "more asynchronous" given that both origins of replication (O_N _and O_J_) are located in the A+T-rich region (rather than at 3/4 of the genome, as in the strand-displacement model of replication) [1], and synthesis of the major coding strand begins after 97% of the minor strand has been replicated [1,2,51]. Being the N-strand, during its single-stranded status, more prone to deaminations than double stranded DNA, A→G and C→U transitions are more likely to occurr. This mutational pressure generates inequalities in the nucleotide composition between the two strands with positive AT-skew and CG-skew values resulting on the J-strand [20,47].

Remarkable strand compositional biases have been previously observed in the mtDNA of some basal hexapod lineages [31,47,52]. In the J-strand of the *C. antarcticus *cytosines are always more frequent than guanines, leading to a positive CG-skew (0.148). Conversely, the AT-skew is only moderately positive, as calculated over the entire genome (0.058) and in III codon positions of the J-strand PCGs (0.048), or negative, as calculated over entire PCGs (-0.082 and -0.27 for protein coding genes oriented on the J-strand and N-strand, respectively). On the other hand, remarkable negative values of AT and CG skew are observed on the N-strand (AT-skew = -0.27 and CG-skew = -0.193, for all PCGs positions; AT-skew = -0.227 and CG-skew = -0.189, for 3^rd ^positions only). This latter bias may be due to the high frequency of mutational events leading to deamination of A and G nucleotides of the displaced N-strand, occurring during the replication of mtDNA. Different rates of transitions (A→G < C→U; [44]) may have eventually led to the asymmetry (negative AT and CG skews) of nucleotide composition and to the higher T% *vs *A% of PCGs oriented on the N-strand.

**Figure 3:**
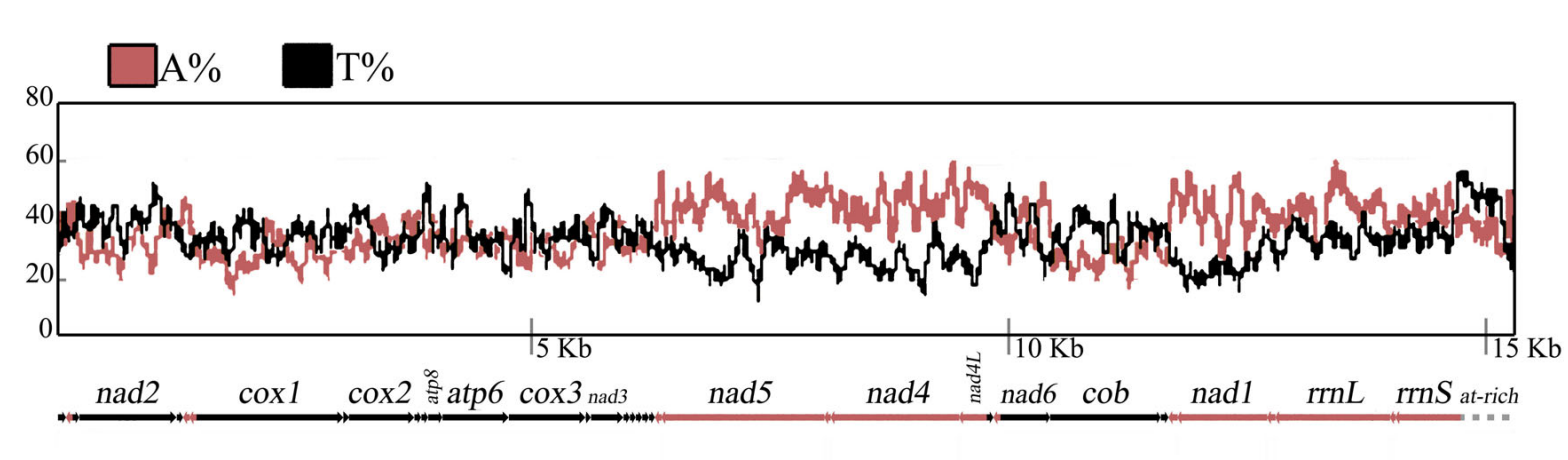
**Graphical representation of the percentage of As (red) and Ts (black) across the whole mtDNA segment calculated using the program MacVector^® ^7.2.3 (Accelrys).** Y-axis values represent nucleotide %, calculated with a 100-bp sliding window; x-axis values represent the nucleotide positions corresponding to the linearized genome. Below the graphs, position and orientation of each gene.

In this respect, the frequency of adenines and thymines is approximately equal in the genes encoded on the J-strand, whereas a higher percentage of A *vs *T (counting on the J-strand) is observed in those oriented on the N-strand (Figure 3). If the strand displacement model of mtDNA replication described in mammals and *Drosophila *also holds for Collembola, we can speculate that the block of genes spanning from *rrnS *to *trnF *(all genes oriented on the N-strand except for *trnT*, *nad6*, *cob *and *trnS(uga)*; Figures 1 and 3) is more prone to the deamination type C→U than the opposite strand (due to higher D_SSH_), and that transitions between C and T are more frequent than those between A and G. Perhaps this trend, observed only in the PCGs oriented on the N-strand, is dependent on a higher number of mutations occurring during transcription, rather than replication, and is consequently less etched in the PCGs encoded on the J-strand. In addition, it has been demonstrated that cytosine is the most unstable of the four bases and the number of C deaminations is more pronounced in single-strand DNAs [53].

**Figure 4:**
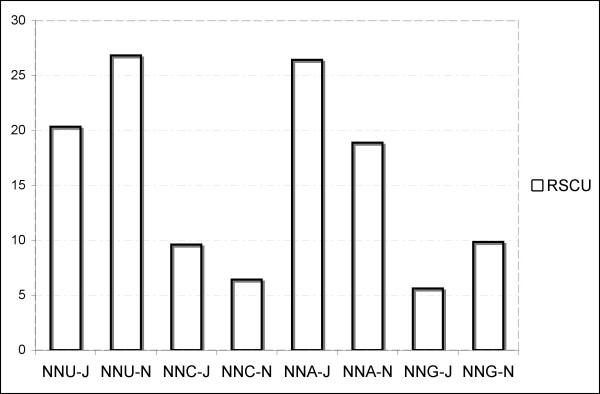
**Across-strand (N & J) comparison of frequencies of codons ending with each nucleotide.** Values on the y-axis represent the sum of RSCU values of codons ending with the same nucleotide across all codon families.

The asymmetrical directional mutation pressure [54] observed in the two strands strongly influences the codon usage of PCGs oriented in opposite directions [55-57]. In this respect, NNA and NNC codons are more frequent than NNU and NNG in the PCGs encoded on the J-strand, whereas the N-strand genes show exactly the opposite trend (Figure 4). Codon usage may also be influenced by other molecular processes such as translational selection efficiency and accuracy [58], which apparently have a stronger influence in organisms with rapid growth rates [59]. In addition, recent analyses of codon usage in four-fold degenerate codons of mammal and fish mtDNAs have demonstrated that there are context-dependent mutational effects (correlations between pairs of neighboring bases). In this respect, the second codon position base strongly influences the presence of a specific nucleotide at fourfold degenerate sites, and these latter are not independent from the first-position nucleotide of the following codon [60].

### Nucleotide variability of collembolan mitochondrial PCGs

The nucleotide variability of each mitochondrial PCG has been estimated calculating gene-by-gene average values of genetic distances (Appendix 1: dist.jpg), across all collembolan taxa for which full (or nearly complete) mtDNAs are available (Appendix 2: list.exl). Distances values are higher for *nad2*, *nad3 *and *cob*, suggesting these genes to be the least conserved ones in the mtDNA of Collembola. Conversely, the more conserved mitochondrial genes are some of the cytochrome family (complexes 1 to 3) and *nad1*. The distribution of the observed nucleotide variability is somewhat difficult to explain. However, according to the proposed model of mtDNA replication mechanism (studied for a restricted number of hexapods taxa [1]), the genes located in the vicinity of the A+T-rich region remain for a longer time in a single-strand state during the mtDNA replication and therefore are more exposed to hydrolytic and oxidative damages [50]. This interpretation could explain the high levels of divergence observed for *nad2*. In addition, sites for the attenuation of the mtDNA transcription mechanism [43] (i.e. located in the vicinity of *nad3 *and *cob*) may also be important to increase the mutation load for neighbour genes. Collectively, these DNA fragments are probably more prone to higher substitution rates than the others due to their position along the mtDNA, although no experimental study has been performed to sustain this hypothesis. Alternatively, structural constraints at the protein level may result in site-specific selective pressures acting differently and in a scattered manner along the mitochondrial genome, with some genes bearing a higher mutational load than others.

## Conclusion

The description and analysis of the complete mtDNA genome sequence of *C. antarcticus *has led to new insights on the mitogenomics of the group, and significantly added to our knowledge on mitochondrial genomes from species adapted to the Antarctic terrestrial ecosystem. Several peculiarities, such as nucleotide and strand-specific compositional bias, the occurrence of truncated stop codons of PCGs and the presence of mismatches and/or incomplete tRNA arms, have been observed. However, no distinguishing molecular features can be associated with adaptation to the extreme polar environment. The gene order is canonical, but two unusual and large non-coding regions were observed in sites of abrupt shift of the coding polarity. Such intergenic spacers may contain the regulatory signals involved in the replication and/or transcription of the mtDNA, although additional data will be needed to better clarify their function. Segments of these intergenic regions can apparently be folded in typical cloverleaf structures, and probably represent duplicated vestigial versions of at least one tRNA gene. This feature seems to be shared by other collembolan mtDNAs, suggesting that the gene junction *trnS(uga)/cob *may represent a "hot spot" for gene rearrangements in Collembola.

## Methods

### Molecular techniques

Specimens of *C. antarcticus *were sampled from Killingbeck I. (Antarctic Peninsula: S 67° 32'; W 68° 7') during the 2002 polar expedition of BAS in collaboration with PNRA. Samples were frozen in liquid nitrogen and conserved al -80°C. Total DNA was extracted using the Wizard SV Genomic Purification System (Promega). The complete mitochondrial genome was sequenced by combining direct sequencing of three PCR fragments amplified with universal primers, and shotgun sequencing of three fragments obtained through Long-PCR amplification with specific primers. Initial amplification and sequencing of three short mtDNA fragments (*cox1/cox2*, *cob *and *rrnL*, totalling ≈ 3 kb) was performed using primers C1-J-1751/C1-N-2191, CB-J-10933/CB-N-11367 and LR-J-12887/LR-N-13398 [61] using several DNA extractions from different *C. antarcticus *specimens. Six specific primers were designed to amplify (using an additional DNA extraction from a single specimen) the rest of the genome in three fragments of approximately 2 kb (1), 6 kb (2) and 4 kb (3) *via *Long-PCR: 1) CANcox2-3453J (CGCTTACTGGATGTAGACAATCGCACAG)/CANcox3-5245N (GAGCCGTACGCTGAGTCTGAAATTG), 2) CANcox3-5269J (CAATTTCAGACTCAGCGTACGGCTC)/CANcob-11444N (CACAATTGTTAAAATTTGTCCCAC) and 3) CANtrnSuga-11574J (AGTGATTAAGCACTTACCTTGAAAGCAAGCTAC)/CANcox1-3053N (CTAGAAGAGGAGAAGCTGCGTTTTGG). Long PCR conditions were the following: 1) fragment CANcox2-3453J/CANcox3-5245N, 94°C for 1 min, 60 for 1 min and 68° for 2 min 30 sec, 35 cycles; 2) fragment CANcox3-5269J/CANcob-11444N, 94°C for 1 min, 50 for 1 min and 68° for 7 min, 35 cycles; 3) fragment CANtrnSuga-11574J/CANcox1-3053N, 94°C for 1 min, 50 for 1 min and 68° for 8 min 30 sec, 35 cycles.

Amplifications were performed on a Gene Amp^® ^PCR System 2700 (Applied Biosystem) in 25 μl reaction volume composed of: 10.75 μl of sterilized distilled water, 2.5 μl of LA PCR Buffer II (Takara), 2.5 μl of 25 mM MgCl_2_, 4 μl of dNTPs mix, 1.25 μl of each primer (10 μM), 2.5 μl of DNA template and 0.25 μl (1.25 U) of TaKaRa LA Taq polymerase (Takara). Long-PCR fragments were purified using the Microcon^® ^Centrifugal Filter Unit (Millipore), randomly sheared to 1.2–1.5 kb DNA segments using a HydroShear device (GeneMachines). Sheared DNA was blunt end-repaired at room temperature for 60 min using 6 U of T4 DNA Polymerase (Roche), 30 U of DNA Polymerase I Klenow (NEB), 10 μl of dNTPs mix, 13 μl of 10× NEB buffer 2 (NEB) in a 115 μl total volume and gel purified using the Wizard^® ^SV Gel and PCR Clean-Up System (Promega). The resulting fragments were ligated into the SmaI site of a pUC18 cloning vector using the Fast-Link DNA ligation Kit (Epicentre) and electroporated into One Shot^® ^TOP10 Electrocomp™ *E. coli *cells (Invitrogen) using standard protocols. Each resulting clone was sequenced on both strands in a CEQ 8000XL automated DNA Analysis System (Beckman Coulter). Eventually, regions of "unsatisfactory coverage" and small gaps in the sequences have been amplified and sequenced using standards protocols and different Long-PCR amplifications as template. Sequences were manually corrected and assembled with the software Sequencher 4.4.2 (Gene Codes). Long-PCR products were composed with those initially generated for short fragments to provide the complete genome sequence. Hence, the final assemblies were based on a minimum sequence coverage of 5×, that is the results of a pool of individuals, although only for a very short portion of the molecule (essentially limited to small fragments of *cox1/cox2 *and *cob*, given the supposed conservation of the amplified region of the *rrnL*). Screening of nucleotide sequences, obtained from specimens of the same locality, provides no extreme variability (essentially limited to the 3^rd ^codon positions) for either *cox1/cox2 *and *cob *fragments (data not shown).

### Gene annotation and analysis

Genes encoding proteins, rRNAs and tRNAs were identified according to their amino acid translation or secondary structure features, respectively. Individual gene sequences were compared with the homologous sequence of other collembolan species available in GenBank and inspected for the presence of gene overlaps, non-canonical start codons and truncated termination codons, and unusual structures at gene junctions. The secondary structure of tRNA genes was manually reconstructed and rendered using the software Rna Viz 2.0 [62]. Nucleotide variability of collembolan mtDNA (see Additional file 1: dist.jpg) was assessed using thirteen alignments of the PCGs. Basic sequence statistics and genetic distances among collembolan genes were calculated using PAUP* 4b8 [63] (see Additional file 2: list.xls), whereas codon usage and RSCU values were calculated using codonw [64]. Strand asymmetry was measured using the formulas AT-skew = [A%-T%]/[A%+T%] and CG-skew = [C%-G%]/[C%+G%] [46,47]. The presence of repeated sequences within non-coding fragments was studied using the mreps software [65]. The presence of additional repeats and palindromic sequences was investigated using Blast, and hits with e-value < 0,05 were retained for further analysis.

## Abbreviations

*atp6 *and *atp8*: genes for ATP synthase subunits 6 and 8; *cox1-3*, genes for subunits I-III of cytochrome *c *oxidase; *cob*: gene for cytochrome *b*; *nad1-6 *and *nad4L*, genes for subunits 1–6 and 4L of NADH dehydrogenase; *rrnL *and *rrnS*: genes for the small and large subunits of ribosomal RNA; *trnX*: genes encoding transfer RNA molecules with corresponding amino acids denoted by the one-letter code and anticodon indicated in parentheses (*xxx*) when necessary; tRNA-X: transfer RNA molecules with corresponding amino acids denoted with a one-letter code; bp: base pair; D_SSH_: single-stranded state of a site on the heavy-strand of mitochondrial DNA; mtDNA: mitochondrial DNA; PCR: Polymerase Chain Reaction; PCG: Protein Coding Gene; RSCU: Relative Synonymous Codon Usage; BAS: British Antarctic Survey (UK); PNRA: Italian National Program of Antarctic Research (I).

## Competing interests

The authors declare that they have no competing interests.

## Authors' contributions

AC sampled the specimens, sequenced the new mitochondrial genome, performed the molecular analyses and drafted the manuscript. SC collaborated in the shotgun sequencing. PC directed the scientific expedition, and collaborated in the sampling of specimens and in the drafting of the manuscript. FN participated in the shotgun procedure and in the analysis of the molecular data. He also critically revised the first draft of the manuscript. FF directed the research and collaborated with the drafting of the final manuscript. All authors read and approved the final manuscript.

## Supplementary Material

Additional file 1 Graphical representation of the average nucleotide genetic distances calculated for every PCGs (y-axis) among the nine collembolan species (but, ten sequences) for which is available a complete (or almost complete) mtDNA. Genetic distances were calculated from 13 independent alignments (performed adjusting preliminary automated alignments obtained using the software RevTrans [66]). The proportion of unalignable (green) positions for each gene-based alignment (left side of x-axis) is depicted. Genetic distances (right side of x-axis) were calculated under the Maximum Likelihood method. Model selection was performed gene-by-gene using an identical tree adapted after [9]. The GTR+I+Γ always resulted as the best fitting model (plus parameters used to accommodate rate heterogeneity among sites), with the only exception of *atp8 *(HKY+I+Γ). Note that the proportion of the alignable sites for this latter gene is very low (57/189), so that in this case the high average values of genetic distances (*) can not be considered reliable, likewise to previous analysis of vertebrate mitochondrial genomes that also described the *atp8 *as the fastest-evolving mtDNA gene [50]. Click here for file

Additional file 2 List of complete (or almost complete) mtDNA collembolan species included in the analysis of gene-by-gene genetic distances with the corresponding GenBank accession number. Click here for file
