# Supplementary figures and images for: The complete mitochondrial genome of the Antarctic springtail Cryptopygus antarcticus (Hexapoda: Collembola)
Source: BMC Genomics. 2008 Jul 1;9:315. doi: 10.1186/1471-2164-9-315 (PMC2483729; doi:10.1186/1471-2164-9-315)

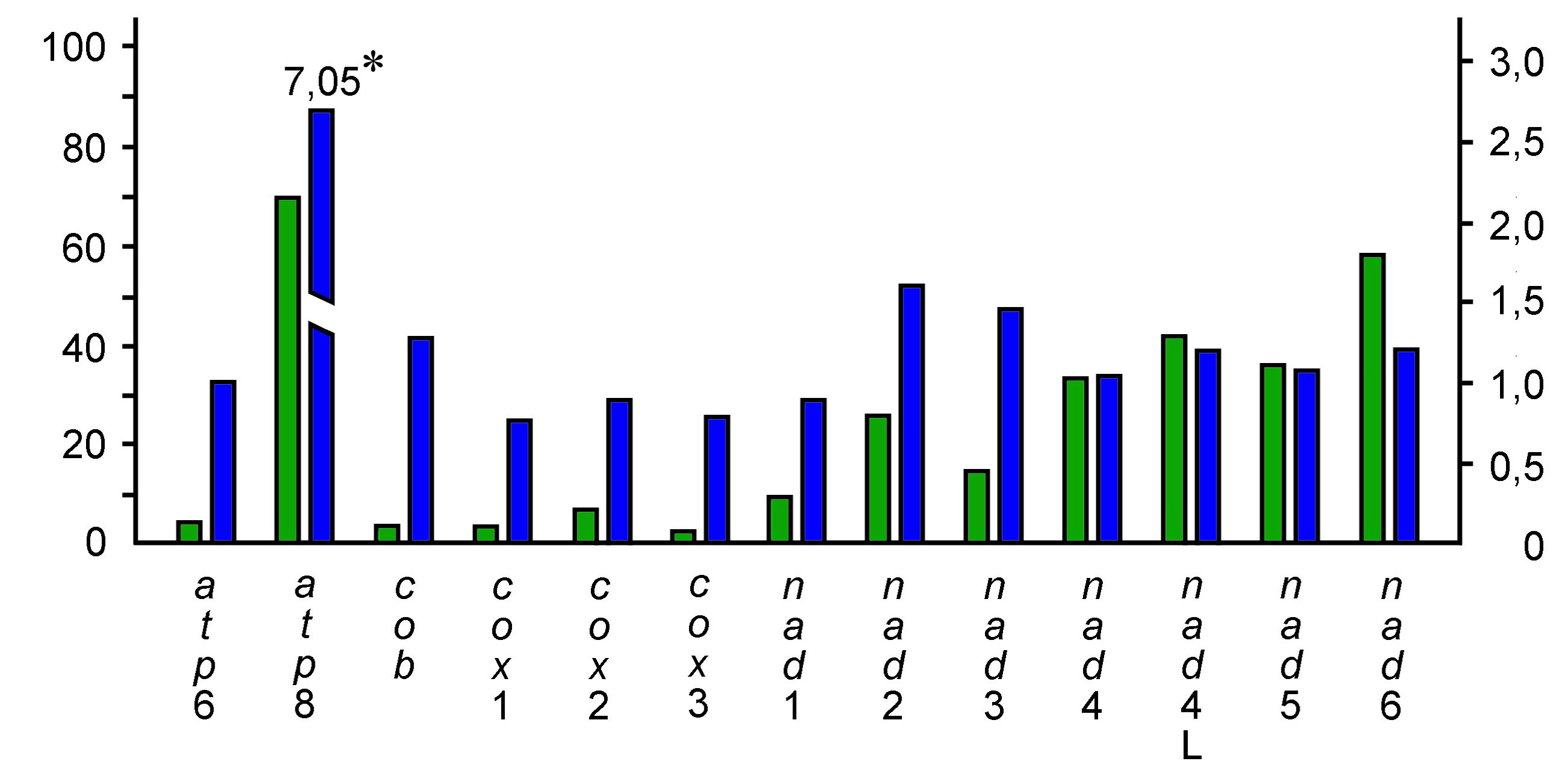

Supplement: Additional file 1 — Graphical representation of the average nucleotide genetic distances calculated for every PCGs (y-axis) among the nine collembolan species (but, ten sequences) for which is available a complete (or almost complete) mtDNA. Genetic distances were calculated from 13 independent alignments (performed adjusting preliminary automated alignments obtained using the software RevTrans [66]). The proportion of unalignable (green) positions for each gene-based alignment (left side of x-axis) is depicted. Genetic distances (right side of x-axis) were calculated under the Maximum Likelihood method. Model selection was performed gene-by-gene using an identical tree adapted after [9]. The GTR+I+Γ always resulted as the best fitting model (plus parameters used to accommodate rate heterogeneity among sites), with the only exception of atp8 (HKY+I+Γ). Note that the proportion of the alignable sites for this latter gene is very low (57/189), so that in this case the high average values of genetic distances (*) can not be considered reliable, likewise to previous analysis of vertebrate mitochondrial genomes that also described the atp8 as the fastest-evolving mtDNA gene [50]. [file 1471-2164-9-315-S1.jpeg]
